# Specialist Insect Herbivore and Light Availability Do Not Interact in the Evolution of an Invasive Plant

**DOI:** 10.1371/journal.pone.0139234

**Published:** 2015-09-25

**Authors:** Zhijie Zhang, Xiaoyun Pan, Ziyan Zhang, Kate S. He, Bo Li

**Affiliations:** 1 Ministry of Education Key Laboratory for Biodiversity Science & Ecological Engineering, Institute of Biodiversity Science, Fudan University, Shanghai, People’s Republic of China; 2 Department of Biological Sciences, Murray State University, Murray, Kentucky, United States of America; Helmholtz Centre for Environmental Research (UFZ), GERMANY

## Abstract

Release from specialist insect herbivores may allow invasive plants to evolve traits associated with decreased resistance and increased competitive ability. Given that there may be genetic trade-off between resistance and tolerance, invasive plants could also become more tolerant to herbivores. Although it is widely acknowledged that light availability affects tolerance to herbivores, little information is available for whether the effect of light availability on tolerance differ between the introduced and native populations. We conducted a common garden experiment in the introduced range of *Alternanthera philoxeroides* using ten invasive US and ten native Argentinean populations at two levels of light availability and in the presence or absence of a specialist stem-boring insect *Agasicles hygrophila*. Plant biomass (total and storage root biomass), two allocation traits (root/shoot ratio and branch intensity, branches biomass/main stem biomass) and two functional traits (specific stem length and specific leaf area), which are potentially associated with herbivore resistance and light capture, were measured. Overall, we found that *A*. *philoxeroides* from introduced ranges had comparable biomass and tolerance to specialist herbivores, lower branch intensity, lower specific stem length and specific leaf area. Moreover, introduced populations displayed higher shade tolerance of storage root biomass and lower plastic response to shading in specific stem length. Finally, light availability had no significant effect on evolution of tolerance to specialist herbivores of *A*. *philoxeroides*. Our results suggest that post-introduction evolution might have occurred in *A*. *philoxeroides*. While light availability did not influence the evolution of tolerance to specialist herbivores, increased shade tolerance and release from specialist insects might have contributed to the successful invasion of *A*. *philoxeroides*.

## Introduction

Rapid adaptation to the novel environments in introduced ranges is one of the mechanisms by which invasive species could spread and become extremely successful invaders after a lag time [1]. A relevant hypothesis is the evolution of increased competitive ability (EICA) hypothesis [2] which posits that release from specialist enemies may have caused evolution in exotic plants, specifically lowered resistance to herbivores and increased growth and competitive ability.

In addition, attempts to explain rapid evolution in invasive species have led to several other hypotheses. A potentially essential but still understudied mechanism is phenotypic plasticity, the property of a genotype to express different phenotypes in different biotic or abiotic environments [3]. Two types of plasticity that are generally considered to be important for the success of invasive plants are (1) the capacity of plants to alter biomass allocation and functional traits in response to different levels of resource availability, and (2) herbivory tolerance, the capacity of plants to reduce the negative effects of damage on fitness [4, 5].

Empirical tests have been strongly biased toward examining the evolution of herbivory resistance rather than tolerance in invasive plants [6, 7]. A trade-off between resistance and tolerance has come to light recently [4, 8]. Therefore, introduced populations of exotic plants are expected to have higher tolerance to specialist herbivores [9]. However, comparisons of herbivory tolerance between native and introduced populations of invasive plants have shown conflicting results, some invasive plants have evolved increased tolerance to specialist insects [10–12]; others have showed no differences in tolerance between native and introduced populations [12, 13]. While these studies have been focused on the novel biotic condition: release from specialist enemies, invasive species can also experience novel resource availability (e.g. light, water and nutrients) [14], which may lead to evolution in functional traits, and influence plant responses to herbivory [15].

Several hypotheses have been proposed to explain the effects of resource availability on tolerance. The compensatory continuum hypothesis (CCH) predicts that herbivory tolerance should be greater in high-resource and low-competition conditions [16]. The growth rate model (GRM), makes the opposite prediction: herbivory tolerance should be greater in more stressful conditions [16, 17]. Most recently, the limiting resource model (LRM) has been proposed. LRM is more flexible than either the CCH or GRM, because it specifically considers which factors are limiting plant fitness and which resources are affected by particular herbivore [18]. Several studies in invasion research have proposed that release from natural enemies and resource availability may interact [19], and then could affect the evolution of anti-herbivore defense [20]. Therefore, we suppose resource availability may also influence the evolution of tolerance in alien invasive plants.

Light is the most important resource for plant performance [21] and previous studies have demonstrated that many exotic plants are likely to invade deeply shaded environment and establish denser stands [22–24]. Therefore, it is suggested that invasive plants may have evolved to be more shade tolerant [25]. Nonetheless, little information is available for whether introduced populations have higher shade tolerance than the natives [25]. In addition, plant responses to herbivores and shading may be in opposition for particular traits [26]. Previous tests have also found light availability affects tolerance to herbivores [26, 27]. Therefore, light availability may affect the evolution of tolerance to specialist herbivores in invasive species.

In this study, we manipulated *Alternanthera philoxeroides* (alligator weed) to detect the effect of light availability on tolerance of plants from two geographical origins (native and invasive ranges). As one of the most rapidly spreading plant invaders, *A*. *philoxeroides* grows abundantly in habitats ranging from open waterways to shaded sites under dense vegetation in the introduced ranges [23, 28] and is reported to be shade tolerant. We have been studying defense against the specialist enemy *Agasicles hygrophila* and competitive ability in invasive *A*. *philoxeroides*. We have found that one aspect of resistance to stem-boring *A*. *hygrophila* is the specific stem length (SSL), calculated as the main stem length divided by the main stem dry weight. We found different genotypes of *A*. *philoxeroides* vary widely in SSL, and genotypes with lower SSL experience much more herbivory (e.g. higher rate of feeding, oviposition and pupation from *A*. *hygrophila*) [29].

Here, we addressed the possible evolution of defense traits and tolerance to *A*. *hygrophila*, and the impact of shade on possible evolution of herbivory tolerance in *A*. *philoxeroides*. We conducted a common garden experiment in the introduced range of *A*. *philoxeroides* using ten invasive (USA) and ten native (Argentina) populations at two levels of light availability and in the presence or absence of a specialist insect *A*. *hygrophila*. Plant biomass (total biomass and storage biomass), two allocation traits (root/shoot ratio and branch intensity, branches biomass/main stem biomass) and two functional traits (specific stem length and specific leaf area) were measured. These traits are all potentially plastic in response to herbivory and shade.

We focused on testing the following effects of each trait: 1) significant effects of plant origin could indicate differences between native and introduced ranges; 2) significant effects of origin by shading interaction on biomass would show differences in shade tolerance between plant origins; 3) significant effects of origin by shading interaction on allocation and functional traits would show differences in plasticity to shading between plant origins; 4) significant effects of origin by herbivore interaction on biomass can indicate differences in herbivory tolerance due to plant origin; 5) significant interactions of origin, shading, and herbivory can indicate the difference in effect of light availability on tolerance between native and introduced populations.

Specifically, we tested the following hypotheses: 1) introduced populations will have lower levels of resistance traits (lower SSL and higher SLA) and enhanced tolerance to *A*. *hygrophila*; 2) introduced populations will be more shade tolerant; 3) the interactive effect of light and herbivory on traits may be different between introduced and native populations.

## Materials and Methods

### Study species

Alligator weed (*Alternanthera philoxeroides*) is a perennial herbaceous plant native to South America from Buenos Aires Province, Argentina (39°S) to southern Brazil (18°S). It typically emerges from belowground buds on storage roots in spring and then spreads vegetatively throughout a growing season to form dense monospecific stands. It overwinters with storage roots and rhizomes [30]. Although plants may produce viable seeds, sexual reproduction contributes little to population regeneration because of both extremely low seed outputs and low germination rates [31, 32]. Vegetative propagation with storage roots and stem fragments is the primary regenerative mode in the field [31]. Biomass allocation to storage roots plays important roles in the life history of *A*. *philoxeroides* [30]: the large root reserves act as the primary energy sources for shoot emergence in early spring and also enable rapid shoot regrowth after herbivory or other physical damage. Root fragments also act as propagules for long-distance dispersal by flooding or human activities (e.g. soil transportation and dredging). Therefore, the biomass of storage roots is in some sense equivalent to the reproductive output for *A*. *philoxeroides* [32].

In the United States, *A*. *philoxeroides* was first introduced in Alabama in 1897 probably through ballast water [33]. Since 1930s, its populations have spread rapidly into northeastern wetlands and also expanded into California [33]. In South America, as many as 40 insect species were recorded on *A*. *philoxeroides*, five of which are considered to be specialists [34]. There are few native insects feeding on introduced *A*. *philoxeroides* in the USA [34], suggesting that release from specialist insect attack could play an important role in explaining its successful invasion. Although three insect species, *Agasicles hygrophila* Selman &Vogt (Coleoptera: Chrysomelidae), *Amynothrips andersoni* O’Neill (Thysanaptera: Phlaeothripidae), and *Arcola malloi* (Pastrana) (Lepidoptera: Pyralidae, Phycitinae), have been introduced into the United States as biological control agents since 1964–1971 [35], these insects are still restricted to certain areas of the ranges of *A*. *philoxeroide*s. Consequently enemy release may continue to act on the host in the US range, but vary regionally in its strength [29]. In turn, this could lead to high inter-population variation or high phenotypic plasticity in defense against specialist herbivores in introduced populations.

**Fig 1:**
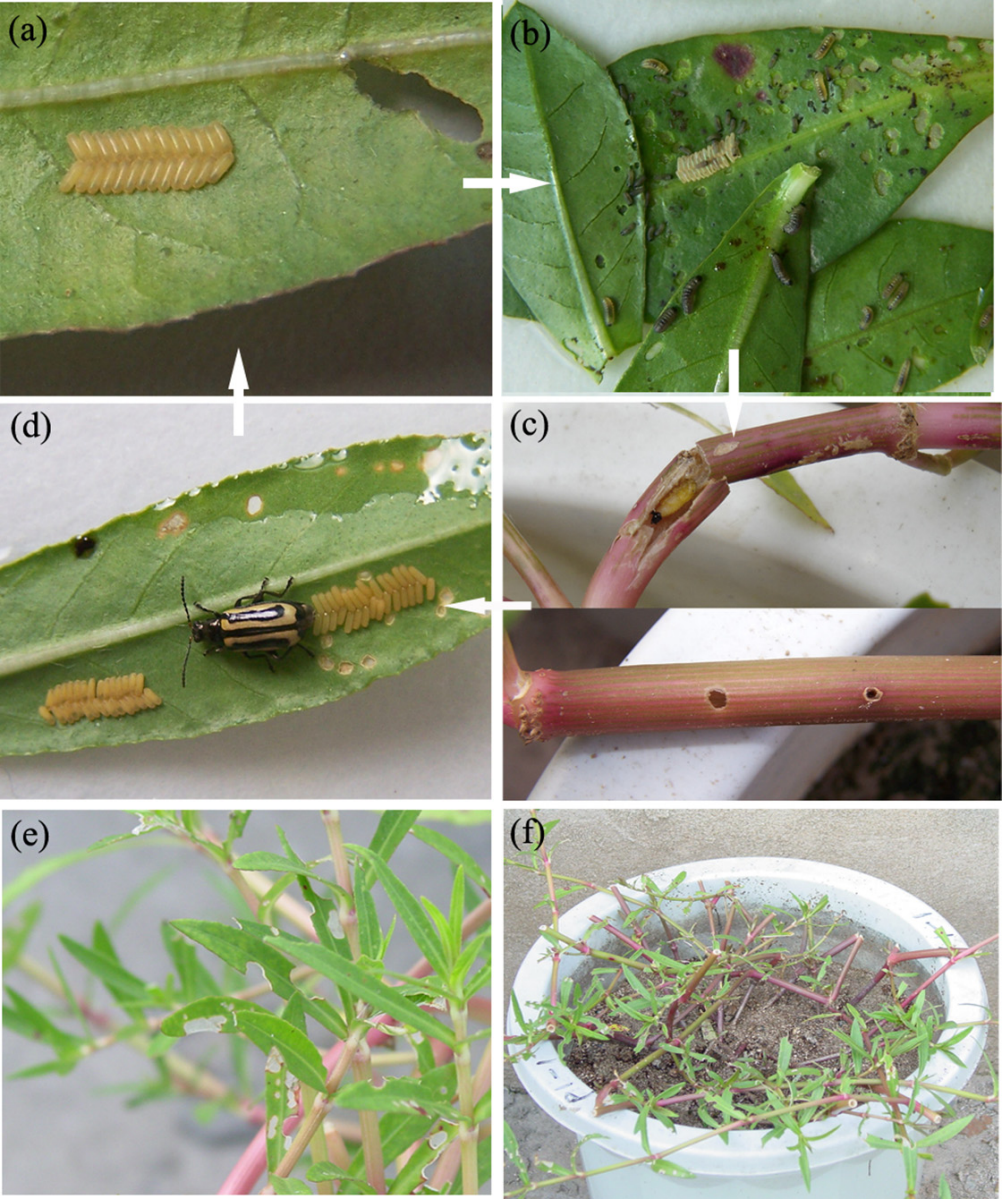
Four life stages of *Agasicles hygrophila* and damage to on *Alternanthera philoxeroides* by *Agasicles hygrophila*. (a) egg stage, 4.1±0.77 days, (b) larva stage, 7.8±0.78 days, (c) pupa stage,8.6±0.57 days, and (d) adult stage, 50.3± 2.42 days [38]. (e) leaf feeding by larvae and adults, (f) stem boring after pupation. Average means ±1 standard error.

Alligator weed flea beetle (*Agasicles hygrophila*) is a specialist phytophagous insect of alligator weed that has been used as one of the most important biological control agents of *A*. *philoxeroides* in USA since 1964. The life stages of *A*. *hygrophila* consist of the egg, 3 larval instars, the pupa, and the adult [34] (Fig 1). Both adults and larvae feed on the leaves and other aerial portions of alligator weed. Mature larvae bore into the hollow stems where they pupate as adults, and exacerbate the acquisition of nitrogen by phloem, which causes damage to the fitness of *A*. *philoxeroides* [34]. It has been suggested that the completion of *A*. *hygrophila*’s life cycle requires hollow stems for pupation, which may contribute, in part, to its specific adaptation to *A*. *philoxeroides* [36, 37].

In the present study, *A*. *hygrophila* was initially introduced from Florida, USA in 1986. We collected insects from the field station of Institute of Plant Protection (Fujian Academy of Agricultural Sciences), Fuzhou, China.

### Seed collection and experimental set-up

In 2006, clones (stem fragments) of *A*. *philoxeroides* weed were collected from ten native populations in Argentina that covered a broad range distribution (Table 1). For each population, we created a mixture of clones from 20–30 maternal families. In 2005, similar collections were made in ten American populations. The sampled stem fragments were transplanted in a greenhouse and vegetatively propagated three times before experiments to remove maternal effects.

**Table 1:** Sampling locations of *Alternanthera philoxeroides*.

Collection site	Latitude	Longitude	Habitat	abundance of *Agasicles hygrophila*
*Argentina (Native)*				
Formosa	25.46'S	58.34'W	wetland	middle
Tucuman	26.54'S	65.18'W	roadside	high
San ignacio	27.11'S	55.53'W	rocks	low
Chaco	27.12'S	59.29'W	shrub vegetation	middle
Misiones	27.15'S	60.31'W	roadside	middle
Corrientes	27.59'S	56.20'W	roadside	middle
Santa Fe	29.16'S	59.49'W	river bank	low
Buenos Aires	34.36'S	58.37'W	pond side	middle
Buenos Aires	35.00'S	58.44'W	roadside	middle
Buenos Aires	37.11'S	59.03'W	roadside	middle
*USA (Introduced)*				
North Carolina	35.42'N	76.42'W	wetland	none
Kentucky	35.11'N	86.22'W	lake bank	none
Arkansas	34.42'N	92.18'W	river bank	none
Georgia	34.12'N	84.06'W	wetland	none
Mississippi	33.31'N	88.28'W	river bank	none
Mississippi	33.16'N	88.47'W	river bank	none
Texas	29.54'N	93.57'W	wetland	none
Louisiana	29.06'N	90.08'W	wetland	none
Florida	26.35'N	81.30'W	river bank	none
Texas	26.12'N	97.38'W	wetland	none

We performed a common garden experiment on the campus of Fudan University, Shanghai, China. The climate is humid subtropical, with rainfall averaging 1895 mm year^–1^, and temperatures averaging 4.2°C in January and 27.9°C in July. The native ranges of *A*. *philoxeroides*, such as Buenos Aires, is humid subtropical, with rainfall averaging 1214 mm year^–1^ and averages 7.4°C in July and 30.4°C in January. The introduced ranges of *A*. *philoxeroides*, for example, the gulf of Mexico, is humid subtropical or humid tropical, with rainfall averaging 1418 mm year^–1^ and averages 10.4°C in January and 28.9°C in July (data from http://worldweather.wmo.int/en/home.html). Therefore, the climates of different origins are similar.

On 18 June 2013, we propagated over 30 stem fragments (with one node, 2cm in length) from one genotype of each population, and 16 randomly selected ramets with similar height and leaf numbers (four leaves) from each population were transferred individually to 700ml plastic pots. Each plant was watered 100ml every two days and received a total of 2 g slow-release 16:9:12 N:P:K fertilizer (Osmocote). A three-factor, randomized block design was then used for executing this experiment. The layout consisted of four blocks. In each block, treatments were randomly assigned to each pot and included two levels of light and two levels of herbivory. Thus, a total of 320 plants were tested in the experiment (2 origins: native and introduced ranges x 2 light x 2 herbivory x 10 populations x 4 blocks).

The two light treatments were control light and shading. Plants in the control light treatment were covered with nylon nettings to exclude herbivores. Plants in the shading treatment were first covered with the nylon nettings and then covered with black fiberglass shading cloth in order to decrease light quantity without changing light quality. The control treatment received approximately 80% light compared to ambient sun measured by an illuminometer (Li-1400, Li-Cor Inc., Lincoln, NB, USA). The shading treatment received approximately 25% light compared to the control light treatment.

Before the herbivory treatment, *A*. *hygrophila* were fed on Fujian populations of *A*. *philoxeroides* and raised for three generations to reduce maternal effects. Four weeks after transplantation, plants in the control treatment received no herbivory. Plants in herbivory treatment were first uncovered from the nettings and shade cloth, then two randomly chosen second-instar larvae were carefully placed on a fully expanded leaf (second or third position from the tip) with a paintbrush, finally the nettings and shade cloth were replaced. The larvae fed on the plants for five days and consumed an average of 4.6% of leaf area in control light conditions and 15.6% of leaf area in shade conditions. Larvae were then allowed to bore into the stem till the end of experiment.

### Data collection

All plants were harvested on 18 August 2013 (six weeks after transplantation). We separated each plant into leaf, main stem, lateral branch, storage root, and fine root. We also measured the length of the main stem, and determined leaf areas with a leaf-area meter (Li-3100, Li-Cor Inc., Lincoln, NB, USA). All plant materials were dried at 70°C to a constant weight (for 72 hours) and weighed to determine leaf dry weight, stem dry weight, branch dry weight, storage root dry weight, and fine root dry weight.

The following allocation parameters were further derived from the above measurements: (1) biomass traits including total biomass as total dry weight, storage root biomass as storage root dry weight; (2) biomass allocation parameters including root/shoot ratio (RSR, [root dry weight]/[shoot dry weight], branch index (BI, [branch dry weight]/[stem dry weight]), (3) functional traits related to light capture and resistance including specific stem length (SSL, [length of stem, cm]/[stem dry weight, g]), and specific leaf area (SLA, [leaf area, cm2]/[leaf dry weight, g]).

### Statistical analyses

We used mixed model ANOVAS (SPSS Proc GLM; v 19.0, SPSS institute Inc, 2010) to test effects of origin (native versus invasive), shading, herbivory, and their interactions on biomass, allocation parameters, and functional traits. We treated population nested within origin as a random effect. We used K-S test and Levene’s test to analyze normality and homoscedasticity respectively. To achieve normality and homoscedasticity, total biomass, BI and SSL were square-root transformed; SLA was log-transformed. In the cases where transformations were done, the results are presented as back-transformed means and standard errors. We used type-III sum of squares for the calculation of F statistics. Tukey’s *post hoc* tests were used to contrast specific means.

## Results

Both of the experimental treatments employed in this study strongly affected the biomass, biomass allocation and functional traits of *Alternanthera philoxeroides* (Table 2), but their interactions were not significant.

**Table 2:** ANOVA showing effects of origin,shading, herbivore, and their interactions on *Alternanthera philoxeroides* according to F ratio.

		Biomass		Allocation parameters		Functional traits	
Source	d.f.	Total	Storage root	RSR	BI	SSL	SLA
origin	1,18	0.16	1.36	0.39	6.75*	4.95*	5.11*
Shading	1,282	1943***	907***	826***	136***	502***	381***
herbivory	1,282	5.71*	2.27	0.003	8.59**	0.47	5.71*
S x H	1,282	1.26	0.175	0.05	3.09	0.021	0.51
O x S	1,282	1.37	5.582*	1.60	0.224	7.85**	3.6
O x H	1,282	0.13	0.00	1.45	0.05	1.29	0.18
O x S x H	1,282	0.12	0.002	0.13	0.03	0.383	2.16

The allocation parameters are: LWR, leaf weight ratio; SWR, stem weight ratio; BWR, branch weight ratio; SRWR, storage-root weight ratio; and FRWR, fine-root weight ratio. The functional traits are: RSR, root/shoot ratio; BI, branch intensity; SSL, specific stem length; and SLA, specific leaf area. Statistical significance is indicated as:

*p<0.05,

**p<0.01,

***p<0.001.

### Differentiation between native and introduced populations

**Fig 2:**
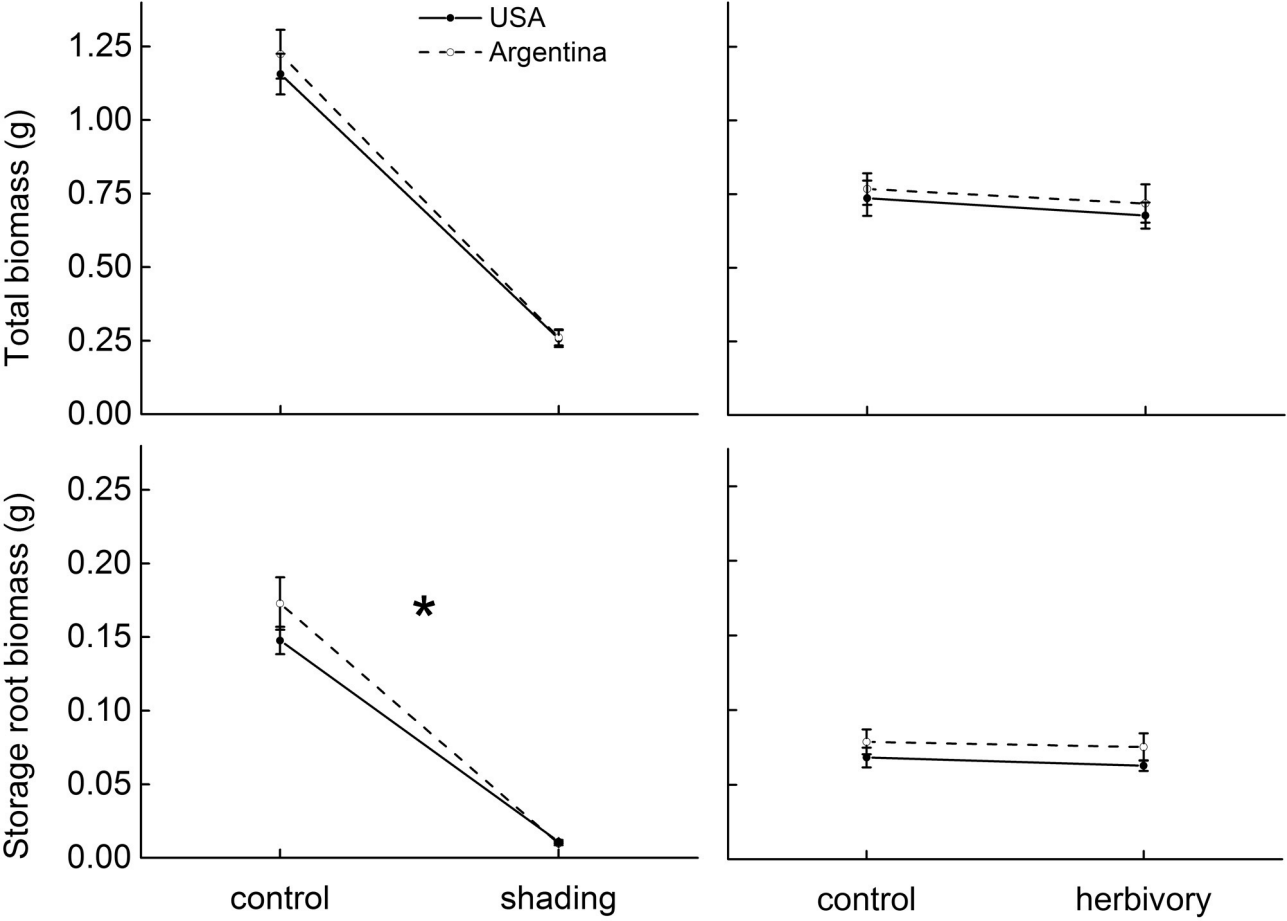
The effects of shading and a specialist herbivore (*Agasicles hygrophila*) on biomass of native (Argentina, dashed lines) and invasive (USA, solid lines) populations of *Alternanthera philoxeroides*. The biomass parameters are: total biomass and storage root biomass. Estimated marginal means ±1 standard error. Statistical significance in origin × treatment is indicated as: *p<0.05.

**Fig 3:**
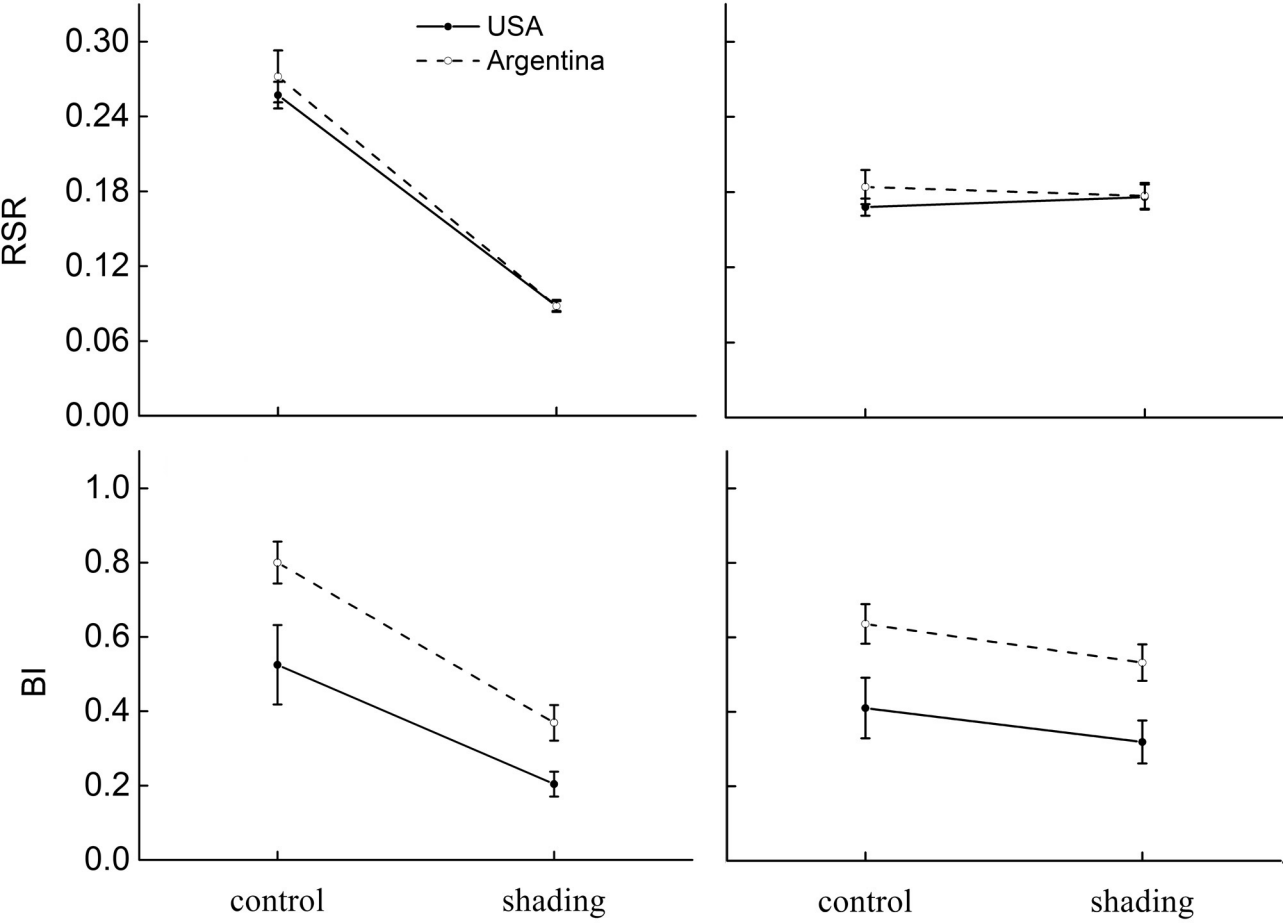
The effects of shading and a specialist herbivore (*Agasicles hygrophila*) on biomass allocation of native (Argentina, dashed lines) and invasive (USA, solid lines) populations of *Alternanthera philoxeroides*. The allocation parameters are: root/shoot ratio,RSR; branch intensity, BI. Estimated marginal means ±1 standard error. There is no statistical significance in origin × treatment.

**Fig 4:**
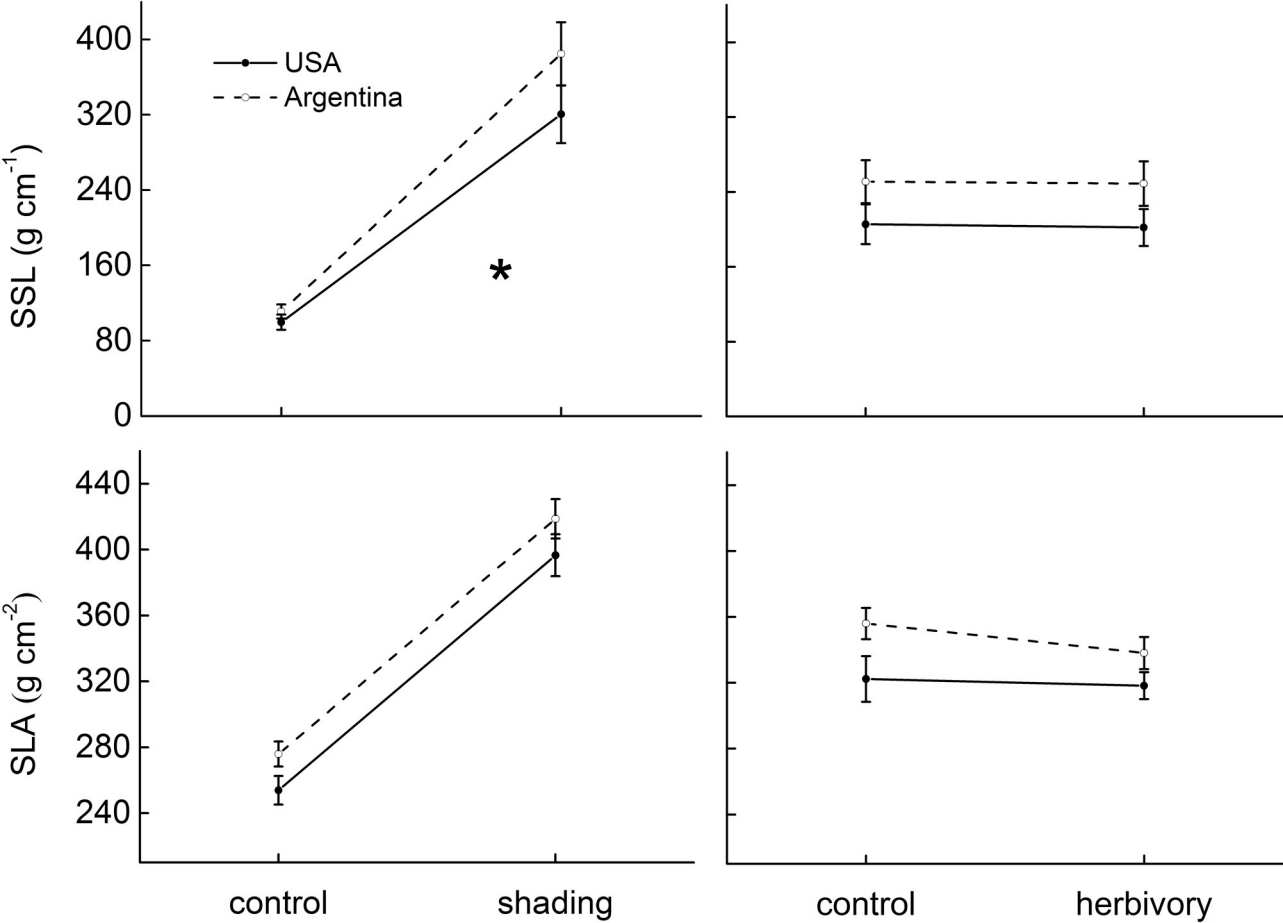
The effects of shading and a specialist herbivore (*Agasicles hygrophila*) on functional traits of native (Argentina, dashed lines) and invasive (USA, solid lines) populations of *Alternanthera philoxeroides*. The functional traits are: specific stem length, SSL; and specific leaf area, SLA. Estimated marginal means ±1 standard error. Statistical significance in origin × treatment is indicated as: *p<0.05.

On average, and across treatments, introduced *A*. *philoxeroides* populations had similar biomass to that of the natives (Table 2, Fig 2). The introduced populations also had lower branch intensity (BI, -23.2%), specific stem length (SSL, -15.3%) and specific leaf area (SLA, -6.2%) than the natives (Table 2, Figs 3 and 4).

### Responses to shading

Shaded plants had significantly lower average total biomass (-77.8%) and storage root biomass (-93.2%) (Table 2, Fig 2), also differed substantially from control plants in biomass allocations and functional traits, with a strong induction of the shading-avoidance response (Table 2, Figs 3 and 4). Shaded plants had lower root/shoot ratio (-65.4%) and branch intensity (-56.8%) (Table 2, Fig 3), but had increased specific stem length (SSL, +235.7%) and specific leaf area (+ 56.3%) (Table 2, Fig 4).

Responses of storage root biomass and SSL to shading differed significantly between native and introduced populations. Storage root biomass was reduced by 70.1% and 75.4% to shading in introduced and native populations, respectively (Table 2, Fig 2). Shading increased SSL by 221.6% and 246.4% in introduced and native populations respectively (Table 2, Fig 4).

### Responses to herbivory and its interaction with light

Plants damaged by specialist *A*. *hygrophila* showed significantly lower total biomass (-6.8%) (Table 2, Fig 2). Herbivory also significantly decreased branch intensity (-18.6%) and specific leaf area (-3.5%) (Table 2, Figs 3 and 4). There was no significant effect of herbivory by shading interaction on all traits measured (Table 2, Figs 2, 3 and 4).

There were no significant origin by herbivory interactions or origin by shading by herbivory interactions for any measured traits (Table 2, Fig 2). The results were virtually identical when we used the percentage of foliar damage as a covariate.

## Discussion

### Differences in biomass allocation and functional traits between native and invasive ranges

Specific stem length (SSL) is a putative structural defense trait of *A*. *philoxeroides* against its stem-boring enemy *A*. *hygrophila* because low SSL stems experience much more herbivory from *A*. *hygrophila* [39]. The lower SSL is indicative of reduced resistance to specific herbivory in introduced *A*. *philoxeroides*. Meanwhile, introduced populations had similar biomass to the natives in the presence or absence of a specialist insect herbivore. These results partially support the EICA hypothesis which predicts that, in the absence of specialist herbivores, introduced plants should reduce allocation to resistance traits but increase biomass production or competitive ability. On the other hand, lower SSL is predicted to reduce plants’ ability to capture light [40]. Thus the significant decrease in SSL may also be indicative of reduced intraspecific competition for light in introduced populations (unpubl. data, X.Y. Pan).

Introduced populations allocated biomass differently than did native populations and showed decreased branch intensity (BI). Plant architectural traits (such as BI) that affect light capture are believed to be vital in determining the degree of self-shading [41] and competitive ability for light [42]. Hence, the decreased BI in the introduced populations is likely to reduce both self-shading and light competition within populations (unpubl. data, X.Y. Pan). These findings could partly explain why invasive plants were often found to form dense and monospecific stands [23].

Specific leaf area (SLA) is a morphological trait which is associated with light capture and resistance to herbivory [43, 44]. Although we found that introduced populations had lower SLA, the fact that they had similar biomass to native populations suggested that light capture may be similar between native and introduced populations of *A*. *philoxeroides*. This is also supported by our previous study which found introduced populations of *A*. *philoxeroides* exhibited similar photosynthetic energy use efficiency and photosynthetic nitrogen use efficiency to the natives [45]. Thus, the lower SLA may be indicative of increased sclerophylly and suggests that the introduced populations of *A*. *philoxeroides* which are not released from generalist herbivores [46] may have higher resistance.

The differences among native and introduced populations in several traits examined in this study suggest that evolution might have occurred in *A*. *philoxeroides* in the introduced ranges. However, we also recognize that this study does not provide direct evidence of such evolutionary. Further studies from population genetic within and between native and introduced populations of *A*. *philoxeroides* are still needed.

### Shade tolerance and plastic responses to shading

*A*. *philoxeroides* responded significantly to shading and displayed shading-avoidance syndrome. Shaded plants showed increased allocation to aboveground tissue, elongation of main stem, high SLA leaves, and decreased allocation to branches, which are similar to the shading responses of many other species [25, 43, 47] and may help *A*. *philoxeroides* to survive in low light environments.

We found no significant origin by shading interaction for total biomass, indicating that native and introduced populations of *A*. *philoxeroides* did not differ in the shade tolerance of total biomass. But we found a significant effect of origin by shading on storage root biomass, and introduced populations showed a flatter slope of the response of storage root biomass to shade, indicating that introduced populations had higher shade tolerance of storage root biomass. Since storage root serves as the primary resource pool from which clonal plants can support population regeneration [30], higher shade tolerance of storage root can enhance the potential for faster future plant regrowth [22]. There is little available literature on comparisons of shade tolerance between native and introduced populations. Matlaga et al.[25] have found that invasive *Miscanthus sinensis* seedlings have similar shade tolerance to natives.

Our result also showed that except for SSL there are no significant differences between native and invasive ranges in plasticity to shading. This is contrary to the hypothesis proposed by Richards et al [48]which predicted increased plasticity in introduced populations, but in accord with other observations. For example, invasive populations of *Microstegium vimineum* showed similar plasticity of SLA to shading [47] and invasive populations of *Clidemia hirta* had similar plasticity to shading for eleven growth and biomass allocation traits [43]. Only one study [49] found leaf area and biomass of *Sapium sebiferum*, showed greater plasticity to light in introduced populations. Given that plasticity is correlated with risks that compromise long-term survival [21, 50] and is not always adaptive [51], the evolution of increased plasticity does not appear to be a universal phenomenon among invasive alien plants. Because plants often increase SSL in response to shading or crowding [40, 52], lower plasticity of SSL to shading in introduced plants may reduce intraspecific competition [53], and hence help to form dense stands in fields.

### Tolerance to herbivory and effect of light

*A*. *philoxeroides* that was damaged by specialist herbivores had a significantly lower total biomass and indicated under-compensating tolerance in our study. We found that damaged plants showed decreased branch intensity (BI). The responses of BI to herbivory are in contrast to several other studies. Bergman found that *Salix caprea* increased branches after foliar damage [54]; Bossdorf et al found that *Alliaria petiolata* increased branches after stem removal [6]. The mechanisms underlying different forms of herbivore tolerance vary widely [5], thus plants may vary in response to different types of damage. Damaged plants showed decreased SLA while shaded plants had increased SLA, indicating plant responses to herbivory and shading may vary among the traits of different functions [26].

Contrary to our predictions, we found no significant herbivory by shading interaction for total biomass or storage root biomass, indicating that light availability did not affect tolerance to specialist herbivores. This finding is inconsistent with the compensatory continuum hypothesis (CCH) or growth rate model (GRM), but seems to be consistent with the prediction of the limiting resource model (LRM) [55]. Studies to date have mixed results. Rogers and Siemann found that *Sapium sebiferum* exhibited equal tolerance at different levels of light availability but *Celtic laevigata* had higher tolerance in high light availability [27]. We also found no significant herbivory by light interaction on resistance-related traits like SSL or SLA, indicating the light availability did not affect the induced resistance in *A*. *philoxeroides*.

In this study, we found no significant difference in tolerance to specialist insects between native and invasive populations, as indicated by the lack of significant herbivory by origin interaction for biomass. This is consistent with previous studies of *Sapium sebiferum* and *Solidago gigantean* [12, 13]. But some alien species have evolved increased tolerance to specialist insects [10–12] in the introduced ranges. Since invaders have no history of local adaptation and co-evolution with the community of enemies in the introduced ranges, their resistance traits may be less effective [5]. Therefore, tolerance is likely to be a more reliable mechanism for reducing the negative effects of damage and should be maintained or increased in the introduced ranges.

To our knowledge, no one has ever tested the effect of light availability on the evolution of herbivore tolerance in invasive plants. Here we have made a first attempt to address this question. We found no significant origin by shading by herbivory interactions for total biomass or storage root biomass, indicating that light availability did not affect the evolution of tolerance to specialist herbivory in invasive populations of *A*. *philoxeroides*. But other resources such as nitrogen and water may drive evolution of tolerance in invasive plants. Future studies need to be done for testing this possibility.

Tolerance in our study may be underestimated given that introduced populations can be less resistant to specialist herbivory [46]. However, as the percentage of foliar damage was not significantly different between native and introduced populations (S1 Fig, stem damage was not available to be measured), tolerance of *A*. *philoxeroides* was likely the result of different biomass allocation patterns.

### Implications for biological control

These results have practical implications for biological control of invasive plants. Although invasive populations of *A*. *philoxeroides* had significantly reduced their resistance to *A*. *hygrophila* [39], maintained tolerance to *A*. *hygrophila* will probably decreased the impact of biological control on introduced populations. Our study may also explain why shading does not accelerate the biological control of *A*. *philoxeroides* using *A*. *hygrophila*. It is important to detect which resources influence plant tolerance of herbivory and to consider these in biocontrol programs.

## Supporting Information

S1 Fig Average of percentage leaf area eaten by *Agasicles hygrophila* on native (Argentina, white) and introduced (USA, black) *Alternanthera philoxeroides*. Estimated marginal means ±1 standard error. P = 0.141. For each seedling we selected all leaves to estimate the magnitude of herbivory. Each of the five leaves was assigned to one of the following categories of damage, based on visual inspection of leaf area removed: 0, no damage; 1, less than 25% damage; 2, from 25% to 50% damage; 3, from 50% to 75% damage; and 4, damage above 75%. The score of all leaves was used to calculate an individual index of herbivory, IH = Σ nC_0–4_N^−1^; where C is the category of damage, n is the number of leaves in the Cth category, and N is the number of total leaves of each plant.
(TIF)
