# Correction: Specialist Insect Herbivore and Light Availability Do Not Interact in the Evolution of an Invasive Plant

**DOI:** 10.1371/journal.pone.0142428

**Published:** 2015-11-04

**Authors:** Zhijie Zhang, Xiaoyun Pan, Ziyan Zhang, Kate S. He, Bo Li

Fig 3 is incorrect. The authors have provided a corrected version here.

**Fig 3:**
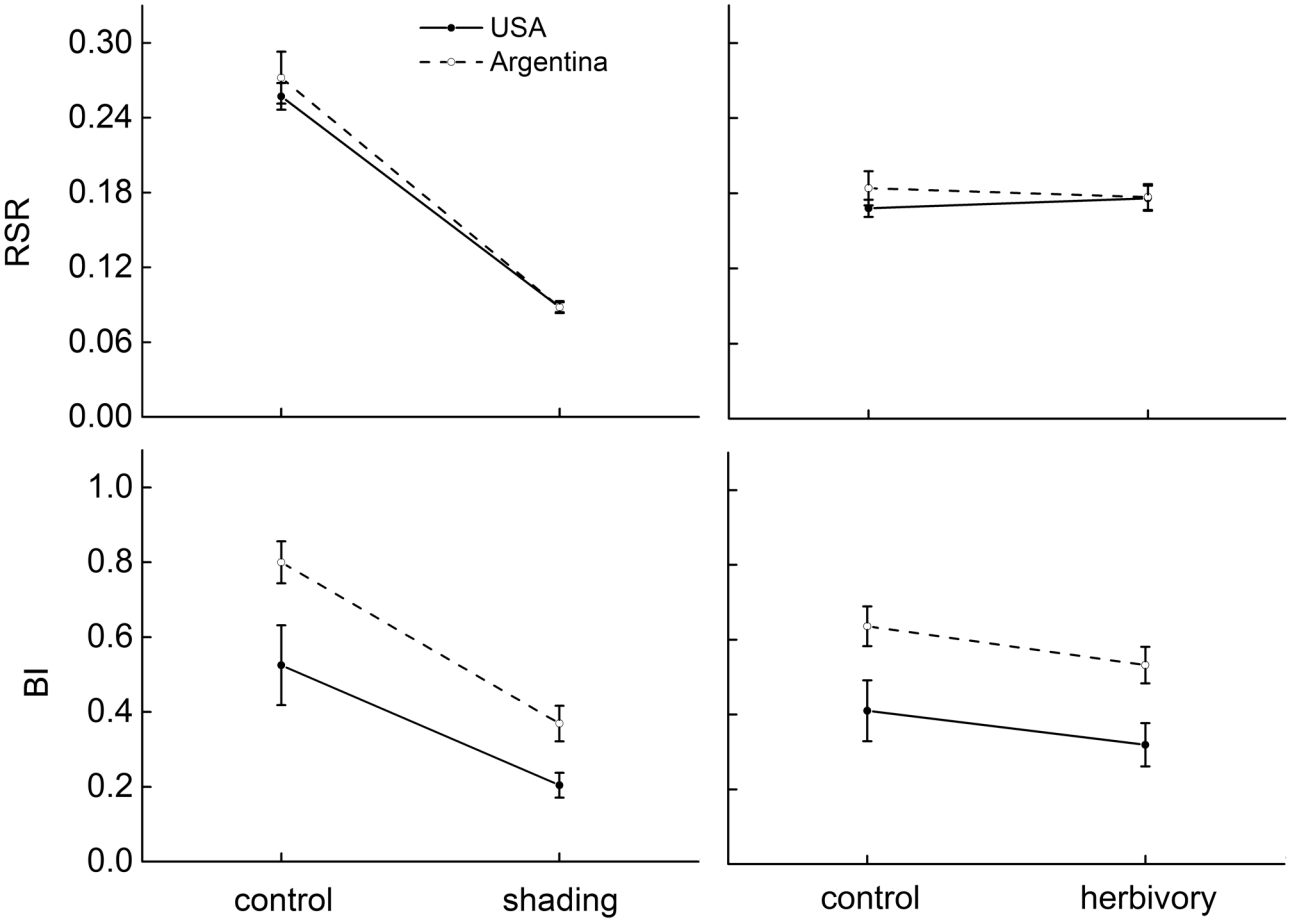
The effects of shading and a specialist herbivore (*Agasicles hygrophila*) on biomass allocation of native (Argentina, dashed lines) and invasive (USA, solid lines) populations of *Alternanthera philoxeroides*. The allocation parameters are: root/shoot ratio,RSR; branch intensity, BI. Estimated marginal means ±1 standard error. There is no statistical significance in origin × treatment.
