# Supplementary figures and images for: Specialist Insect Herbivore and Light Availability Do Not Interact in the Evolution of an Invasive Plant
Source: PLoS One. 2015 Sep 25;10(9):e0139234. doi: 10.1371/journal.pone.0139234 (PMC4583994; doi:10.1371/journal.pone.0139234)

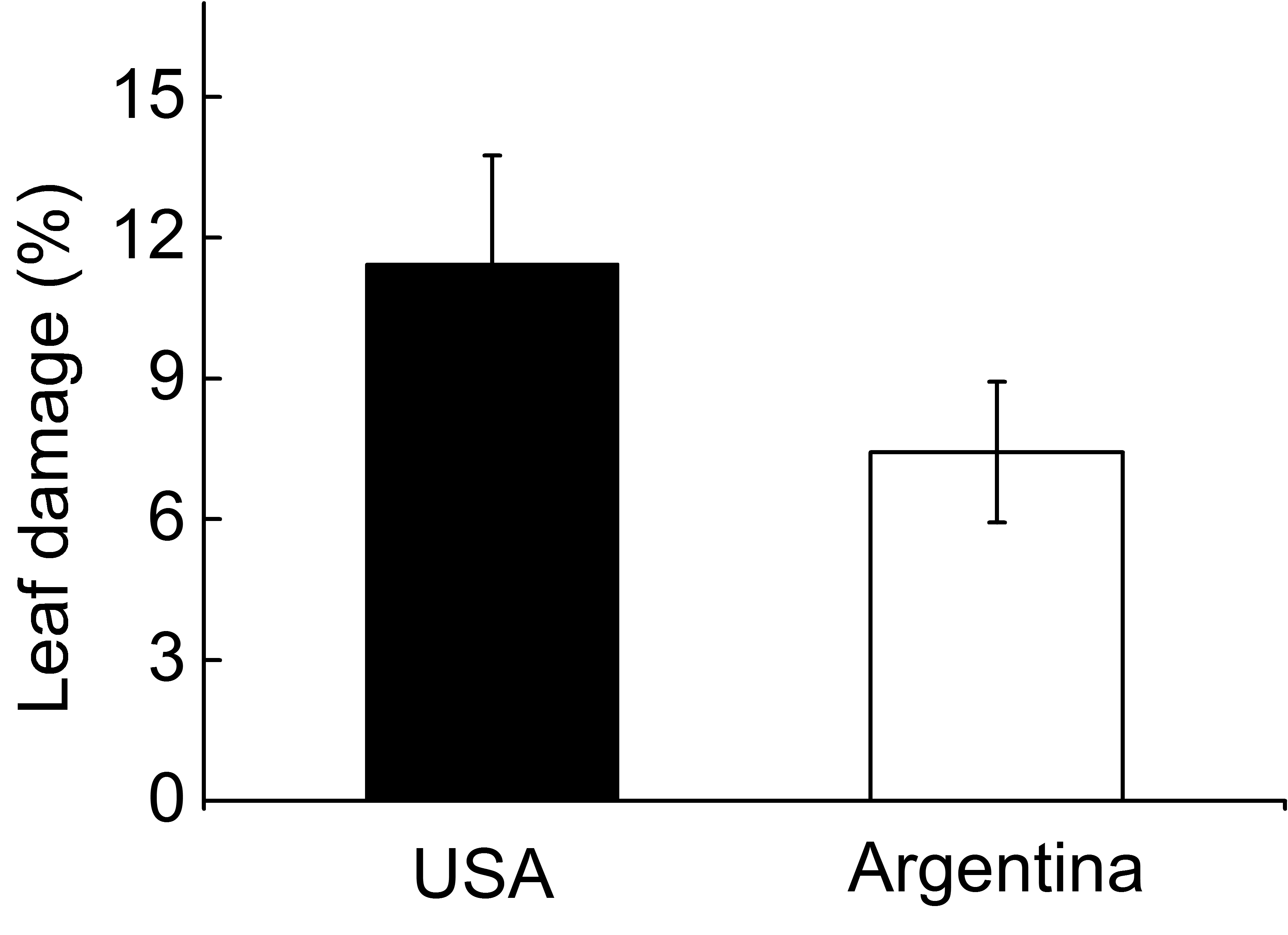

Supplement: S1 Fig — Estimated marginal means ±1 standard error. P = 0.141. For each seedling we selected all leaves to estimate the magnitude of herbivory. Each of the five leaves was assigned to one of the following categories of damage, based on visual inspection of leaf area removed: 0, no damage; 1, less than 25% damage; 2, from 25% to 50% damage; 3, from 50% to 75% damage; and 4, damage above 75%. The score of all leaves was used to calculate an individual index of herbivory, IH = Σ nC0–4N−1; where C is the category of damage, n is the number of leaves in the Cth category, and N is the number of total leaves of each plant. (TIF) [file pone.0139234.s001.tif]
